# Clinical and Molecular Heterogeneity in Patients with Innate Resistance to Anti-PD-1 +/− Anti-CTLA-4 Immunotherapy in Metastatic Melanoma Reveals Distinct Therapeutic Targets

**DOI:** 10.3390/cancers13133186

**Published:** 2021-06-25

**Authors:** Tuba N. Gide, Inês Pires da Silva, Camelia Quek, Peter M. Ferguson, Marcel Batten, Ping Shang, Tasnia Ahmed, Alexander M. Menzies, Matteo S. Carlino, Robyn P. M. Saw, John F. Thompson, Richard A. Scolyer, Georgina V. Long, James S. Wilmott

**Affiliations:** 1 Melanoma Institute Australia, The University of Sydney, Sydney, NSW 2065, Australia; tuba.gide@sydney.edu.au (T.N.G.); Ines.Silva@melanoma.org.au (I.P.d.S.); camelia.quek@melanoma.org.au (C.Q.); Peter.Ferguson@melanoma.org.au (P.M.F.); marcel.batten@sydney.edu.au (M.B.); ping.shang@melanoma.org.au (P.S.); tasnia.ahmed@melanoma.org.au (T.A.); alexander.menzies@sydney.edu.au (A.M.M.); matteo.carlino@sydney.edu.au (M.S.C.); robyn.saw@melanoma.org.au (R.P.M.S.); John.Thompson@melanoma.org.au (J.F.T.); Richard.Scolyer@health.nsw.gov.au (R.A.S.); georgina.long@sydney.edu.au (G.V.L.); 2 Charles Perkins Centre, The University of Sydney, Sydney, NSW 2006, Australia; 3 Sydney Medical School, The University of Sydney, Sydney, NSW 2006, Australia; 4 NSW Health Pathology, Sydney, NSW 2050, Australia; 5 Royal Prince Alfred Hospital, Sydney, NSW 2050, Australia; 6 Royal North Shore Hospital, Sydney, NSW 2065, Australia; 7 Mater Hospital, North Sydney, NSW 2060, Australia; 8 Crown Princess Mary Cancer Centre, Westmead and Blacktown Hospitals, Sydney, NSW 2145, Australia

**Keywords:** immunotherapy resistance, tumour-infiltrating lymphocytes, RNA-sequencing, melanoma, anti-PD-1, anti-CTLA-4

## Abstract

**Simple Summary:**

Immune checkpoint therapies have significantly improved the survival of patients with metastatic melanoma, however approximately 50% of patients exhibit no response. Understanding the underlying clinical, pathologic and genetic factors associated with failed response to immunotherapies is key to identifying therapeutic strategies to overcome resistance. Here, we investigated the baseline tumour characteristics of patients with innate resistance to anti-PD-1-based immunotherapies. This study is the first on non-responders to integrate detailed clinical and molecular analyses and has identified two distinct clusters of patients with clinically relevant key targetable proteins.

**Abstract:**

While immune checkpoint inhibitors targeting the CTLA-4 and PD-1 receptors have significantly improved outcomes of many patients with metastatic melanoma, there remains a group of patients who demonstrate no benefit. In this study, we sought to characterise patients who do not respond to anti-PD-1-based therapies based on their clinical, genetic and immune profiles. Forty patients with metastatic melanoma who did not respond to anti-PD-1 +/− anti-CTLA-4 treatment were identified. Targeted RNA sequencing (*n* = 37) was performed on pretreatment formalin-fixed paraffin-embedded (FFPE) melanoma specimens. Patients clustered into two groups based on the expression profiles of 26 differentially expressed genes: an immune gene rich group (*n* = 17) expressing genes associated with immune and T cell signalling, and a second group (*n* = 20) expressing genes associated with metabolism, signal transduction and neuronal signalling. Multiplex immunohistochemistry validated significantly higher densities of tumour-infiltrating lymphocytes (TILs) and macrophages in the immune gene-rich group. This TIL-high subset of patients also demonstrated higher expression of alternative immune-regulatory drug targets compared to the TIL-low group. Patients were also subdivided into rapid progressors and other progressors (cut-off 2 mo progression-free survival), with significantly lower TILs (*p* = 0.04) and CD68+ macrophages (*p* = 0.0091) in the rapid progressors. Furthermore, a trend towards a higher tumour burden was observed in rapid progressors (*p* = 0.06). These data highlight the need for a personalised and multilayer (clinical and molecular) approach for identifying the most appropriate treatments for anti-PD-1 resistant patients and provides insight into how individual treatment strategies can be achieved.

## 1. Introduction

Immunotherapies targeting the immune checkpoint receptors programmed cell death receptor 1 (PD-1) and cytotoxic T-lymphocyte antigen 4 (CTLA-4) have resulted in significantly improved clinical outcomes in patients with metastatic melanoma [1,2,3] and are now standard of care treatment [4]. However, there remains a subset of patients (approximately 40–65%) who fail to elicit a response to anti-PD-1-based immunotherapies or progress after a period of disease control. These non-responding patients can be categorised into two main groups: (i) Innate or primary resistance representing those who do not respond to immune checkpoint blockade at all or have stable disease for less than 6 months before disease progression (approximately 20–40% of patients), and (ii) Secondary or acquired resistance representing those who relapse and develop disease progression following an initial response to immunotherapies (approximately 20–30% of patients) [5,6]. Patients with innate resistance to immunotherapies are more likely to die from their melanoma compared to patients with acquired resistance, and therefore represent the greatest clinical unmet need. 

Several studies have been conducted investigating the mechanisms associated with innate resistance and potential biomarkers that may be used to discriminate responders from non-responders to anti-PD-1 therapy. The characterisation of melanoma patients into different groups based on the presence of tumour-infiltrating lymphocytes (TILs) and PD-L1 expression predicted that PD-L1^+^TIL^+^ patients were the most likely to respond to immunotherapies, while PD-L1^−^TIL^−^ patients were likely not to respond to single-agent blockade probably due to a lack of pre-existing T cells [7]. Low levels of tumour mutational burden and a low T cell-inflamed gene expression profile were jointly associated with limited clinical responses to anti-PD-1 therapy in multiple cancer types [8]. In melanoma cell lines, JAK 1/2 loss-of-function mutations resulted in a lack of PD-L1 expression following exposure to interferon gamma [9] and in resistance to immunotherapy. The enrichment of transcriptional genes associated with mesenchymal transition, wound healing and angiogenesis were identified in pretreatment melanoma specimens from non-responders to anti-PD-1 monotherapy [10]. In addition, the upregulation of other immune checkpoints such as TIM-3, LAG-3 and IDO has been associated with innate resistance to anti-PD-1-based immunotherapies in melanoma [11,12,13]. The aforementioned studies involved a comparison between responders and non-responders to anti-PD-1 therapies. However, an in-depth characterisation of the profiles of non-responding patients alone has not yet been conducted, particularly with emphasis on patients with rapidly progressive disease.

Previously, we showed that responders to anti-PD-1-based therapies displayed higher densities of effector memory T cells compared to non-responders [14,15], and this was more predictive of response than CD8^+^ or PD-L1^+^ cell densities [15]. In this study, we further characterised melanoma patients with innate resistance to anti-PD-1-based immunotherapies in order to identify possible therapeutic strategies to overcome resistance. Using the data obtained from targeted RNA sequencing and multiplex immunohistochemistry of baseline (pretreatment) melanoma tissue, along with the patient clinical characteristics, we determined the clinical, immune, and genetic profiles that define innate resistance to immune checkpoint therapies.

## 2. Materials and Methods

### 2.1. Patient and Sample Selection

This study was approved by the New South Wales Department of Health Human Research Ethics Committee (Protocol no. X15-0454). All human research procedures were performed according to the National Health and Medical Research Council of Australia guidelines. The study was conducted in accordance with the Declaration of Helsinki. Samples were acquired with patients’ informed consent from the Melanoma Institute Australia Biospecimen bank. The Melanoma Institute Australia’s research database was searched to identify non-responding patients to anti-PD-1 monotherapy and anti-PD-1 combined with anti-CTLA-4, and all available archival formalin-fixed paraffin-embedded (FFPE) biopsies were collected from these patients. Patients with progressive disease (PD) and stable disease (SD) of less than or equal to 6 months based on the RECIST 1.1 criteria [16] were classified as non-responders with innate resistance and were included in this study as per previous studies [6,15]. Patients with innate resistance were further subdivided into “rapid progressors” and “other progressors” based on a 2-month progression-free survival. 

The clinical cohort consisted of 40 non-responders with innate resistance to anti-PD-1-based immunotherapies. Pretreatment metastatic FFPE specimens were available for 36 out of 40 patients and a primary melanoma specimen was included for one patient where no other samples were available. Of the 40 patients, targeted RNA sequencing data were available for 37 patients and multiplex immunofluorescence data was available for 28 patients. 

### 2.2. Assessment of TILs Based on Haematoxylin and Eosin (H&E) Staining

The density and distribution of TILs were scored on H&E stained sections by a histopathologist (P.M.F.) and stratified into three groups, based on previously published methods [17]. Melanomas with low TILs showed an absent or mild focal lymphocytic infiltrate; intermediate TILs, a marked focal, moderate multifocal or a mild diffuse TIL infiltrate; and high TILs, a moderate or marked diffuse TIL infiltrate.

### 2.3. Targeted RNA Sequencing Library Preparation and Sequencing

Specimens were assessed for tumour content based on the H&E sections and manually macrodissected for RNA extraction using the High Pure FFPET RNA isolation kit (Roche Life Science). Library preparation was performed using the QIAseq Targeted RNA Custom Panel (Qiagen) targeting 533 genes (Supplementary Table S1), as per the manufacturer’s guidelines. Briefly, 450 ng of RNA was used for complementary DNA (cDNA) synthesis, followed by a polymerase chain reaction (PCR) for molecular barcoding with the BC primers. The barcoded cDNA was purified and then incubated with the LA and RS2 primers, followed by a universal PCR for library amplification and sample indexing. The final library was quantified using the QIAseq™ Library Quant Assay Kit (Qiagen) and sequenced on an Illumina Nextseq 500. Genes for the custom panel were selected based on the differentially expressed genes previously identified between responders and non-responders to anti-PD-1-based therapies [15], as well as drug targets currently available in clinical trials for the treatment of solid tumours (NCT02608268, NCT02554812 and NCT02668770). 

### 2.4. Targeted Gene Expression Analysis 

Data analysis was performed using the Qiagen GeneGlobe Data Analysis Centre to convert the FASTQ files to Unique Molecular Indices (UMI) counts per gene per patient. Raw counts were filtered to exclude genes with low reads (<5 counts per million in at least 5 samples). Count data were normalised using the *DESeq()* function [18]. Differential gene expression analysis between non-responders based on their levels of TILs (high, intermediate or low), and time to progression (rapid progressor versus other progressor) was performed using the DESeq2 R package [18]. Significantly differentially expressed genes (DEGs) were identified as those with an adjusted *p* value of ≤0.1.

### 2.5. Hierarchical Clustering Analysis of Gene Expression Profiles

Unsupervised hierarchical clustering was performed on the targeted RNA sequencing data using the “Euclidean” distance and “complete” linkage methods. Heatmaps illustrating the different clusters based on gene expression were produced using ClustVis [19]. Supervised clustering for TILs and progression were also performed on the gene expression data using ClustVis [19]. 

### 2.6. Gene Set Enrichment Analysis (GSEA)

Gene set enrichment analysis was performed on a list of the pre-ranked DEGs using GSEA software [20,21]. DEGs were ranked according to their log2 fold change values. The absolute enrichment scores were derived using the C2 curated subset of the Molecular Signature Database version 7.2 [21,22], based on KEGG pathways [23]. The significantly enriched pathways were defined by nominal *p* ≤ 0.1 and FDR < 0.25.

### 2.7. Multiplex Immunohistochemistry (mIHC)

Four micrometre FFPE melanoma sections mounted on Superfrost Plus slides (Thermo-Scientific, MA, USA) were heated in the oven at 65 °C for 20 min, deparaffinised in xylene and rehydrated in graded ethanols. Antigen retrieval was performed in pH9 HIER buffer in the Decloaking Chamber (Biocare Medical, CA, USA) at 110 °C for 10 min. Slides were cooled on the benchtop in TBST for 10 min before commencing staining using an Autostainer plus (DAKO, CA, USA). Tissue sections were blocked with 3% hydrogen peroxide in TBST for 5 min, and then incubated with the antibody for CD68 (Cell Marque, CA, USA; 1:500) for 30 min. The antibody was detected using the Opal Polymer HRP Ms + Rb (Onestep) (AKOYA Biosciences, MA, USA) detection system, before visualisation using Opal520 TSA (1:100) for another 5 min. Subsequently, antigen retrieval was conducted again to prepare the slides for the next antibody. Using this method, all samples were stained sequentially with CD16a (Abcam, Cambridge, UK; 1:400) visualised with Opal620 TSA (1:100), SOX10 (Biocare Medical, 1:200) visualised with Opal690 TSA (1:100), PD-L1 (Cell Signaling Technology, MA, USA; 1:1000) visualised with Opal650 TSA (1:100) and CD8 (Dako, 1:1500) visualised with Opal570 TSA (1:100). Slides were counterstained with DAPI (1:2000) for nuclei visualisation, and cover slipped using the ProLong^®^ Diamond Antifade Mountant (Invitrogen, MA, USA).

### 2.8. Multispectral Image Analysis

Slides were imaged using the Vectra 3.0 and multispectral fluorescent images visualised in Phenochart v.1.0.8 (AKOYA Biosciences). High-resolution images (20×) were captured of the entire tumour and surrounding peritumoural regions. Images were spectrally unmixed in inForm v.2.4.1 (AKOYA Biosciences), and the individual 20× images stitched into a single multispectral image for each tumour specimen for analysis in HALO v.2.2 (Indica Labs, Albuquerque, NM, USA). The Random Forest tissue classifier algorithm was trained to recognise tumour and peritumour based on the presence or absence of SOX10. Positivity for each individual marker was determined by optimised thresholds based on the staining intensity. 

### 2.9. Statistical Analysis

The baseline clinical factors were summarised using frequencies and proportions for categorical variables, and median and range for continuous variables. The Mann–Whitney U test was used to compare continuous variables (tumour burden [sum of diameters of large metastases according to RECIST criteria], number of metastases and number of different sites of disease) between two groups of patients based on the level of TILs (“high/intermediate” TILs versus “low” TILs), progression-free survival (PFS) (PFS < 2 months vs. PFS ≥ 2 months) and based on gene expression (Cluster 1 versus Cluster 2). Fisher’s exact test was used to compare categorical variables (including normal versus elevated LDH, presence versus absence of brain metastases, presence versus absence of liver metastases, BRAF mutation versus wild type, lymph node versus subcutaneous metastases and intermediate/low drug target expression versus high drug target expression). Survival curves were estimated using the Kaplan–Meier method and the log rank test was performed to compare PFS and overall survival (OS) between two groups. All statistical analyses were conducted using the GraphPad PRISM (PRISM Version 8.0d) and R version 3.6.3 (R Foundation for Statistical Computing, Vienna, Austria).

## 3. Results

### 3.1. Patient Characteristics

This study included forty patients with metastatic melanoma (stage IV or unresectable stage III American Joint Committee on Cancer [AJCC], 8th edition [24]) treated with anti-PD-1 alone (nivolumab or pembrolizumab; 65%, 26/40) or in combination with anti-CTLA-4 (anti-PD-1 + ipilimumab; 35%, 14/40), who had progressive disease or stable disease for less than or equal to 6 months (Table 1 and Supplementary Table S2). At baseline, the median age was 67 years (range 34–96), 65% (26/40) were male, 60% (24/40) were AJCC stage M1c/d (28% had liver metastases) and 38% (15/40) had an elevated lactate dehydrogenase (LDH). Thirteen percent of non-responders (5/40) had a BRAF V600E mutation and 33% (13/40) had a NRAS mutation.

### 3.2. Clinical Characteristics of Patients with Different Patterns of Progression

**Figure 1:**
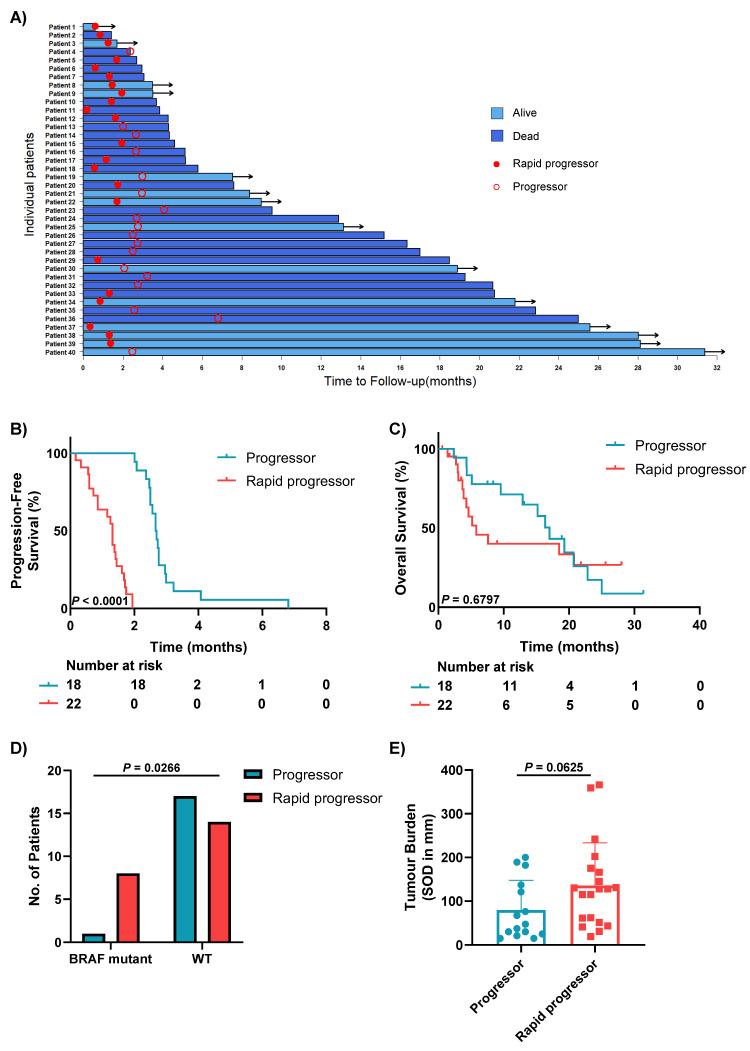
Clinical characteristics of non-responders to anti-PD-1-based therapies. (**A**) Swimmer’s plot illustrating the overall survival of non-responders to anti-PD-1-based therapies. Empty circle—progression; filled circle—rapid progression; dark blue—dead; light blue with arrow—alive. (**B**) Kaplan–Meier curves demonstrating significantly shorter progression-free survival in rapid progressors compared to progressors. (**C**) Kaplan–Meier curves demonstrating no significant difference in overall survival between rapid progressors and progressors. (**D**) Bar graph showing the proportion of progressors and rapid progressors with BRAF mutations, assessed using the Fisher’s exact test. (**E**) Box plot comparing tumour burden (measured as the sum of diameters in mm) between rapid progressors and progressors. Error bars represent mean ± SD.

All patients in the study had progressed on anti-PD-1-based treatment, with a median follow-up of 25.6 months and median PFS of 1.84 months (0.16–6.81 months) at data cut January 2019 (Figure 1A). As per the definition, PFS was significantly shorter for the rapid progressors (patients who progressed in less than 2 months) compared to the other progressors (mPFS 1.3 vs. 2.7 months, *p* < 0.0001; Figure 1B), while the difference in OS was not statistically significant (mOS 5.8 vs. 17 months, 1-year OS 40% vs. 71%, *p* = 0.6797) (Figure 1C). A significantly higher proportion of rapid progressors had a BRAF mutation compared to other progressors (*p* = 0.0266; Figure 1D); 63% (5/8) of these were BRAF V600E mutations (Supplementary Table S3). While there was no difference between rapid progressors and other progressors with regards to clinical variables including baseline age, gender, ECOG PS, LDH, total number and number of different sites of metastases, or presence of brain and liver metastases, there was a trend towards a higher tumour disease burden in the rapid progressors compared to the others (median sum of diameters by RECIST 128mm vs. 47mm, *p* = 0.0625) (Figure 1E and Supplementary Table S3). 

Following progression on anti-PD-1-based therapy, 45% of patients (18/40) did not receive any additional systemic drug treatment. Fifty-five percent (22/40) had subsequent treatments; 13 of these 22 patients (59%) had anti-PD-1 monotherapy and 9 (41%) had combination ipilimumab + anti-PD-1 therapy (Supplementary Table S4). However, there were no significant differences in OS between patients who received subsequent treatments and those who did not (*p* = 0.1797; Supplementary Figure S1).

At data cut, 14 patients (35%) were still alive, and there was a trend towards a lower proportion of patients with liver metastases in this group of patients compared with the patients who have died (*p* = 0.0614). There were no differences between these two groups regarding baseline age, gender, ECOG PS, LDH, tumour burden, total number of different sites of metastases and presence of brain metastases (Supplementary Table S5).

### 3.3. Rapid Progressors Have Lower Expression of TILs and CD68+ Macrophages Compared to Patients Who Progressed after 2 Months 

**Figure 2:**
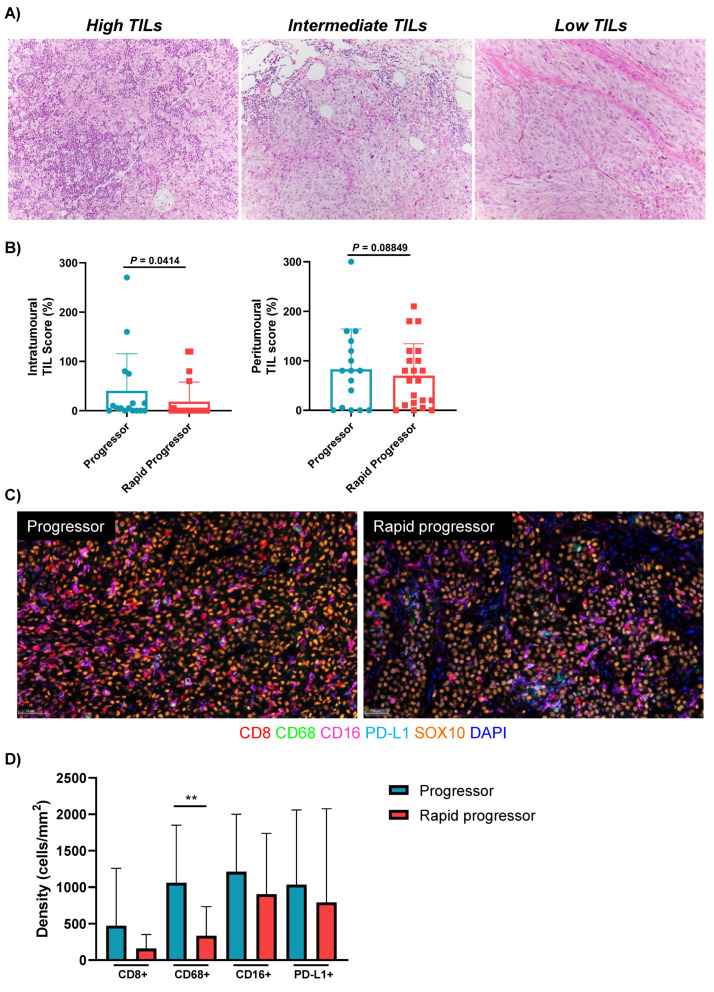
Immune profiles of non-responders to anti-PD-1-based therapies. (**A**) Non-responding melanomas showing variation in the degree of tumour-infiltrating lymphocytes (TILs). Representative photomicrographs demonstrate the three grades of TILs: high TILs—diffuse infiltrates of moderate to marked intensity; intermediate TILs—marked focal, moderate multifocal, or mild diffuse infiltrates; and low TILs—absent or focal mild infiltrates. (**B**) Box plots comparing the intratumoural and peritumoural TIL score between rapid progressors (*n* = 21) and progressors (*n* = 16). (**C**) Representative multiplex immunofluorescent images of intratumoural regions stained with CD8, CD68, CD16, PD-L1, SOX10 and DAPI, from a progressor and rapid progressor on anti-PD-1-based therapy. (**D**) Bar graphs comparing the densities of intratumoural immune cell populations between progressors (*n* = 12) and rapid progressors (*n* = 16). Error bars represent mean ± SD. ** *p* < 0.01.

H&E staining was performed on 37 of the 40 non-responders to anti-PD-1-based therapies (PD1, *n* = 23; IPI+PD1, *n* = 14), and patients were separated into three groups based on their pathological TIL score. Three of the 40 patients were excluded due to unavailability of the appropriate FFPE specimen. Only 4 of 37 (11%) patients had “high” TILs, 19/37 (51%) patients had intermediate TILs, and 14/37 (38%) patients had “low” TILs (Figure 2A). 

In order to assess whether different TIL patterns were associated with different clinical characteristics, the pathological TIL score was used to divide patients into two groups, low TILs versus intermediate/high TILs. No significant differences were observed between the two groups regarding any of the clinical characteristics evaluated, including age, gender, ECOG PS, LDH, tumour burden, total number and number of different sites of metastases and presence of brain or liver metastases (Supplementary Table S6). In addition, there was no significant difference in PFS (*p* = 0.9536; 3-month PFS rate 9% intermediate/high TILs vs. 7% low TILs) or OS (*p* = 0.5086; 1-year OS rate 57% intermediate/high TILs vs. 50% low TILs) between the two groups of patients (Supplementary Figure S2A,B).

We then assessed the level of TILs in the previously defined clinical groups: rapid progressors versus other progressors; and dead versus alive patients. Rapid progressors had a significantly lower abundance of intratumoural TILs compared to patients who progressed after 2 months (*p* = 0.0414; Figure 2B); while no difference was observed in peritumoural TILs between the two groups (*p* = 0.08849). Additionally, there was no significant difference in the level of TILs between patients who were dead or alive at last follow-up (*p* = 0.2595; Supplementary Figure S3).

The immune populations from the mIHC were then examined in progressors and rapid progressors (Figure 2C). Rapid progressors had significantly lower densities of intratumoural CD68+ macrophages compared to other progressors (*p* = 0.0091) (Figure 2D). While rapid progressors also had lower levels of CD8+, CD16+ and PD-L1+ cells, these did not reach significance (*p* > 0.05) in this cohort. 

### 3.4. Differential Expression Analysis Identifies Two Distinct Groups of Non-Responders to Anti-PD-1-Based Therapies

**Figure 3:**
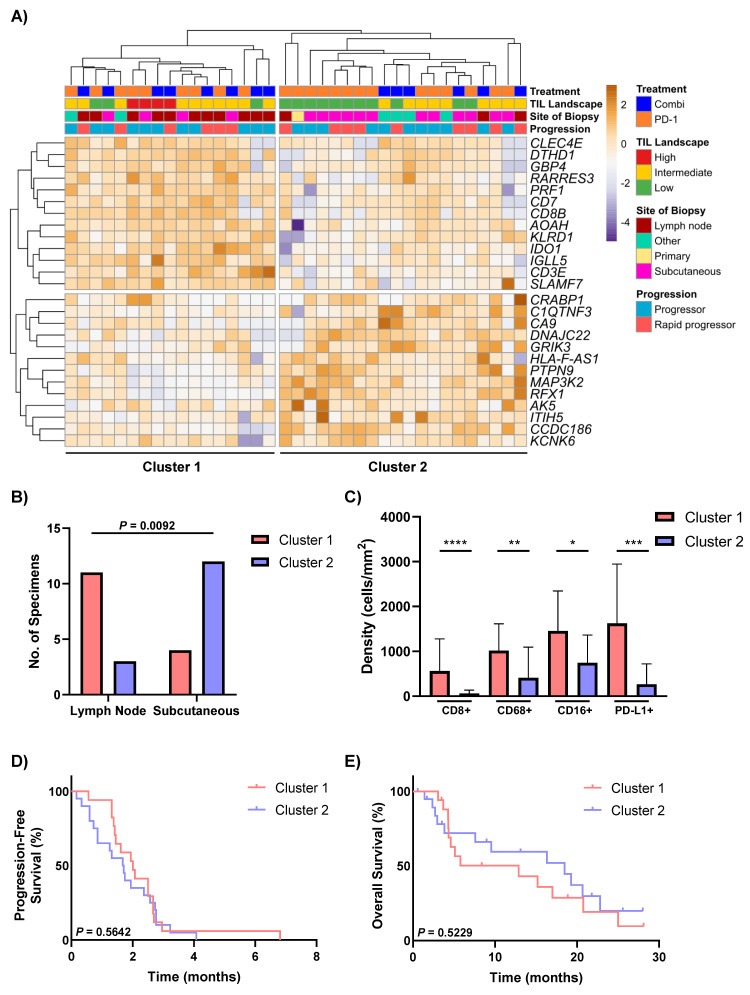
Gene expression profiles of non-responders to anti-PD-1-based therapies. (**A**) Unsupervised hierarchical clustering of 26 differentially expressed genes (DEG) in non-responders to anti-PD-1-based therapies. (**B**) Comparison of the proportions of lymph node and subcutaneous samples between DEG Cluster 1 and Cluster 2, assessed using the Fisher’s exact test. (**C**) Bar graphs comparing the densities of intratumoural immune cell populations (CD8, CD68, CD16 and PD-L1) between patients in DEG Cluster 1 (*n* = 13) and Cluster 2 (*n* = 15). Kaplan–Meier curves illustrating no significant differences in (**D**) progression-free survival or (**E**) overall survival between Cluster 1 and Cluster 2. Error bars represent mean ± SD. * *p* < 0.05, ** *p* < 0.01, *** *p* < 0.001, **** *p* < 0.0001.

Targeted RNA sequencing including 533 genes was completed for 37/40 non-responders to anti-PD-1-based therapies, and differential gene expression analysis was performed to identify differences between groups based on their level of TILs (low, intermediate and high TILs) and PFS (rapid progressors versus the other progressors). Unsupervised hierarchical clustering was performed on 26 DEGs and revealed two distinct clusters of non-responders based on their gene expression profiles and levels of TILs. Cluster 1 (*n* = 17) of non-responders consisted largely of patients with high/intermediate TILs (82%; 14/17) and displayed a higher expression of genes associated with antigen recognition, presentation and processing (*CD8B* and *KLRD1*), the innate and adaptive immune response (*IGLL5, CLEC4E, CD7* and *SLAMF7*), and interferon signalling (*GBP4* and *IDO1*) (Figure 3A). In contrast, the majority of patients in Cluster 2 (*n* = 20) had low TILs (55%; 11/20) and expressed genes associated with hypoxia and metabolism (*ITIH5, CA9, AK5* and *MAP3K2*), signal transduction (*CRABP1* and *DTHD1*) and neuronal signalling (*GRIK3* and *KCNK6*) (Figure 3A). Pathway analysis revealed dysregulation of the hematopoietic cell lineage, primary immunodeficiency and T cell receptor signalling pathway in Cluster 2 (nominal *p* ≤ 0.1 and q < 0.2; Supplementary Table S7).

The proportion of lymph node metastasis specimens was significantly higher in Cluster 1 (65%; 11/17) compared to Cluster 2 (15%; 3/20), which consisted of mostly subcutaneous metastasis specimens (*p* = 0.0092; Figure 3B). Cluster 1 also had significantly higher intratumoural densities of CD8+ T cells (*p* <0.0001), CD68+ macrophages (*p* = 0.003), CD16+ macrophages (*p* = 0.0204) and PD-L1+ cells (*p* = 0.0003) in comparison to Cluster 2 (Figure 3C). No significant differences were observed in PFS or OS (*p* = 0.5642 and *p* = 0.5229, respectively) (Figure 3D,E), or in any of the clinical characteristics between the two clusters (Supplementary Table S8).

### 3.5. Heterogeneous Expression of Immune Drug Targets within Non-Responders to Anti-PD-1-Based Therapies 

**Figure 4:**
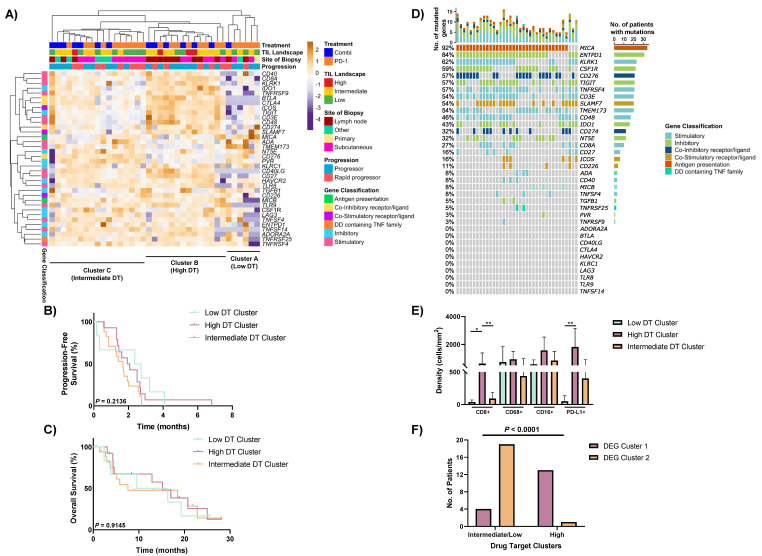
Gene expression of selected drug targets in non-responders. (**A**) Unsupervised hierarchical clustering of gene expression of 36 immune genes including drug targets in non-responders to anti-PD-1-based therapies. Kaplan–Meier curves illustrating no significant differences in (**B**) progression-free survival, or (**C**) overall survival between the three drug target (DT) clusters. (**D**) Oncoplot illustrating the positive expression of drug target genes (log2 counts > 4) in non-responders to anti-PD-1-based therapies. (**E**) Comparison of intratumoural immune populations (CD8, CD68, CD16 and PD-L1) from the multiplex immunohistochemistry between patients in the high DT cluster (*n* = 11), intermediate DT cluster (*n* = 12) and low DT cluster (*n* = 5). (**F**) Bar graph illustrating the number of patients from the intermediate/low DT and high DT cluster in each DEG cluster, assessed using the Fisher’s exact test. Error bars represent mean ± SD. * *p* < 0.05, ** *p* < 0.01.

Next, the expression of 36 druggable immune targets was evaluated via unsupervised hierarchical clustering, revealing three distinct groups of non-responders (Figure 4A). Cluster A (*n* = 6) of non-responders displayed an overall low expression of the various stimulatory and inhibitory immune checkpoint molecules. However, interestingly, all six patients displayed high expression of at least one druggable target, including expression of *TMEM173* (STING) in 4/6 patients, *SLAMF7* in 2/6 patients and *CD274* (PD-L1) in one patient (Figure 4A). Cluster B (*n* = 14) presented with higher expression of the drug targets, including *IDO1, BTLA, ICOS* and *TIGIT*. Finally, Cluster C (*n* = 17) was an intermediate group of patients with heterogeneous expression of the various drug targets.

Cluster A (Low drug target (DT) cluster) had a higher proportion of patients treated with anti-PD-1 alone (83%; 5/6), compared to 57% (8/14) in Cluster B (High DT cluster) and 59% (10/17) in Cluster C (Intermediate DT cluster) (Figure 4A). Sixty-four percent of the specimens in the high DT cluster were lymph node metastasis, while 53% of the intermediate DT cluster were subcutaneous metastasis specimens. Five of six “other” metastasis specimens (including brain and lung) clustered into the intermediate DT cluster. A higher proportion of the rapid progressors were present in the intermediate DT cluster (47%; 8/17), compared to the high (36%; 5/14) and low (33%; 2/6) DT clusters. The low and intermediate clusters also presented with a higher proportion of specimens with low TILs (48%; 11/23), in comparison to the high DT cluster (20%; 3/15). Furthermore, a higher proportion of patients in the intermediate DT group presented with elevated baseline LDH compared to patients in the low and high DT clusters (*p* = 0.0541; Supplementary Table S9). There were no significant differences between the three clusters for either PFS (*p* = 0.2136; Figure 4B) or OS (*p* = 0.9145; Figure 4C).

To further characterise the drug target profiles of the non-responders, we assessed the expression of these drug target genes in each individual patient (Figure 4D). The most highly expressed genes were *MICA* (MHC Class I) (expressed in 92% of patients), and *ENTPD1* (CD39; 84%). Other highly expressed drug targets with agents available in clinical trials included *CD276* (B7-H3) (expressed in 57% of non-responders), *TIGIT* (57%), *TNFRSF4* (OX40; 57%) and *TMEM173* (STING; 54%). In contrast, drug targets including *PVR* (CD155) and *TNFRSF9* (4-1BB) were only expressed in 3% (1/37) of non-responders. Notably, *CD274* (PD-L1) was expressed in 32% (12/37) of non-responders in our cohort, while *CTLA4* was not expressed in any sample.

### 3.6. Drug Target Profiles of Non-Responders Are Associated with TIL Expression and the DEG Clusters

Finally, we investigated the expression of the various immune populations from the mIHC to determine whether there were any associations with the drug target profiles. The high DT cluster expressed significantly higher levels of CD8+ T cells and PD-L1 expression compared to the low DT cluster (*p* = 0.0103 and *p* = 0.0043, respectively) and intermediate DT cluster (*p* = 0.0091 and *p* = 0.0503, respectively) (Figure 4E and Supplementary Figure S4). While the high DT group had a higher CD16+ cell density than the low DT cluster (median 1619 cells/mm^2^ vs. 515 cells/mm^2^), it did not reach significance (*p* = 0.0919). 

Interestingly, all six patients in the low DT cluster and 76% (13/17) of patients in the intermediate DT cluster were in Cluster 2 (high metabolic cluster) from the 533 gene DEG analysis (Figure 4F and Supplementary Figure S4). Conversely, a significantly higher proportion of patients in the high DT cluster (93%; 13/14) grouped into Cluster 1 (higher TILs cluster) from the DEG analysis (*p* < 0.0001; Figure 4F). 

## 4. Discussion

In this study, we investigated the baseline clinical, immune and transcriptomic profiles of patients with metastatic melanoma with innate resistance (no response) to anti-PD-1 monotherapy or combination ipilimumab and anti-PD-1 therapy. Patients with rapid progression (PFS < 2 months) had a significantly lower level of TILs, and a higher rate of BRAF mutations compared to other non-responders (PFS ≥ 2 months). Our study revealed two distinct clusters of non-responders based on the expression of genes involved in various pathways of resistance, with one group displaying higher expression of immune-related genes, and the second group expressing genes involved in metabolism. Importantly, these clusters were not associated with any clinical factors. The high degree of interpatient clinical and molecular heterogeneity observed within the non-responders has implications for the treatment of patients with innate resistance to anti-PD-1-based immunotherapies and suggests they will require personalised management strategies to achieve the best outcomes. 

Previous studies have investigated the clinical factors that may be associated with rapid progression on immune checkpoint therapy [25,26,27,28]; however, many of these results are inconsistent across studies. Our study showed increased rates of BRAF mutation in patients with rapid progression, with a higher proportion of V600E mutations. While BRAF mutations have not previously been associated with rapid progression, increased rates of BRAF mutation in melanoma patients were observed in non-responders to immunotherapy compared to responders [29]. Furthermore, patients with V600K mutant melanomas had significantly higher response rates to anti-PD-1 immunotherapy than patients with V600E mutations [30]. In our cohort, we also observed a lower level of TILs, and a trend towards a higher tumour burden in rapid progressors. Tumour burden was not associated with hyper-progression in any previous studies [25,26,27]. In non-small cell lung carcinoma (NSCLC), low levels of highly differentiated CD4+ T cells were associated with innate resistance and hyper-progression to immunotherapy [31], however in contrast, Russo et al. did not observe any correlation between hyper-progression and levels of CD4+/CD8+ TILs [32]. While a higher tumour burden and lower TILs has previously been shown to be associated with poor response to immunotherapy [10,33,34], our study is the first to demonstrate that these factors, in addition to a BRAF mutation, also discriminate rapid progressors from other progressors in melanoma. The conflicting findings among different studies highlight the critical need for further translational research to define the exact mechanism behind rapid progression on immunotherapy and identify more accurate predictive biomarkers of progression and response to specific agents. 

Tumour-extrinsic resistance factors such as low TILs and altered immune-related gene expression have generally been associated with a poor response to immunotherapy [10,15,35]. In this study, we found that non-responders could also be separated into two groups based on their immune and gene expression profiles: a TIL-high group expressing various immune drug targets, and genes associated with antigen presentation and an immune response, and a TIL-low group expressing genes associated with metabolism and signal transduction, and low expression of alternative drug targets. These findings are in line with our previous study demonstrating low drug target expression in TIL-cold tumours in non-responders to anti-PD-1-based therapies [15]. However, importantly, patients with an overall low drug target profile all expressed distinct therapeutic targets that are currently being tested in clinical trials in melanoma and other cancer types (NCT03329950 [CD40]; NCT03809624 [PD-L1 X 4-1BB]; NCT04148937 [CD73]; NCT04144140 [STING]; NCT03894618 [OX40L]), suggesting different mechanisms of resistance and different treatment options for patients. Therefore, while these clusters were not associated with PFS or OS, our findings provide insight into potential alternative treatments for patients who do not respond to standard-of-care immunotherapies. 

In addition to the interpatient heterogeneity of TIL levels and drug target expression demonstrated in our study, several other studies have described the intrapatient heterogeneity that exists in patients with melanoma [36,37]. Three different resistance-conferring mutations were identified in different metastases from one patient with resistance to a BRAF inhibitor, demonstrating the presence of multiple resistance mechanisms within a single patient [38]. Significant genomic heterogeneity was also observed in all patients (*n* = 15) with multiple melanoma metastases [39], and gene expression signatures were found to vary from a primary melanoma to the corresponding metastasis [40]. Furthermore, we recently demonstrated the intrapatient heterogeneity of IDO1 between primary melanomas, and their corresponding lymph node and distant metastases [41]. These studies suggest that it is likely a number of different molecular factors that contribute to therapy resistance, and highlight the importance of studying clinical and molecular factors together (multilayered approach) in order to make the most informed decision regarding appropriate treatments for non-responding patients. 

The main limitation of this study is the small size of the cohort, which diminished the power to analyse associations between molecular findings and clinical outcomes. However, the study is the largest of its kind to date with a specific focus on highly clinically annotated non-responders to immunotherapy, and to our knowledge, is the first study characterising these non-responders by integrating detailed clinical and molecular data. 

## 5. Conclusions

The general profiles of “hot” and “cold” tumours have been associated with good and poor responses to immunotherapy, respectively. Here, we have shown for the first time that non-responders to immunotherapy constitute a heterogeneous group of patients and are likely to have distinct mechanisms of resistance. We further identified two distinct clusters of patients with clinically-relevant key targetable proteins and highlight the need for a personalised approach in order to achieve precision immunotherapy.

## Figures and Tables

**Table 1:** Baseline patient demographics and clinical characteristics.

Number of Patients, n (%)	40 (100)
**Age** **median (range)**	67 (34–96)
**Sex, n (%)** **Male**	26 (65)
**ECOG, n (%)** **>=1**	19 (48)
**Mutational Status, n (%)** **BRAF-Mutant** **V600E** **Other** **NRAS-Mutant** **WT ^1^**	5 (13) 4 (10) 13 (33) 18 (45)
**LDH, n (%)** **>ULN**	15 (38)
**AJCC v8 ^2^, n (%)** **IIIc/M1a/M1b** **M1c** **M1d**	16 (40) 12 (30) 12 (30)
**Liver Metastases, n (%)**	11 (28)
**Treatment, n (%)** **Anti-PD-1** **Anti-CTLA-4 + Anti-PD-1**	26 (65) 14 (35)
**Toxicity, n (%)** **Any ≥G3**	8 (20)

ECOG—Eastern Cooperative Oncology Group performance status; WT—wild type; LDH—lactate dehydrogenase; ULN—upper limit of normal; AJCC—American Joint Committee on Cancer 8th edition; PD-1—programmed cell death receptor 1; CTLA-4—cytotoxic T-lymphocyte antigen 4; n—number; %—percentage. ^1^ WT also includes mutations in the c-KIT and KRAS genes. ^2^ AJCC v8 anatomic staging, excluding LDH.

## Data Availability

The targeted RNA sequencing data presented in this study are available in the European Nucleotide Archive (ENA) [42], under accession number PRJEB45846.

## Supplementary Materials

The following are available online at https://www.mdpi.com/article/10.3390/cancers13133186/s1, Supplementary Table S1. Genes included in the custom QIAseq targeted RNA panel. Supplementary Table S2. Clinical summary of patients treated with anti-PD-1-based therapies. Supplementary Table S3. Comparison of baseline clinical factors in rapid progressors (PFS < 2 months) versus other progressors (PFS ≥ 2 months). Supplementary Table S4. Subsequent therapies received by patients who progressed on anti-PD-1-based therapies. Supplementary Table S5. Comparison of baseline clinical factors in patients who are still alive versus those who have died at the data cut. Supplementary Table S6. Comparison of baseline clinical factors in non-responders with low TILs versus intermediate/high TILs. Supplementary Table S7. KEGG pathway analysis of differentially expressed genes. Supplementary Table S8. Comparison of baseline clinical factors between non-responders in Cluster 1 and Cluster 2 based on unsupervised hierarchical clustering of 26 differentially expressed genes. Supplementary Table S9. Comparison of baseline clinical factors between non-responders in Cluster A, Cluster B and Cluster C based on unsupervised hierarchical clustering of 36 genes, including druggable immune checkpoints. Supplementary Figure S1. Survival of non-responders based on subsequent therapies received. Kaplan–Meier curve showing the overall survival of non-responders who received subsequent therapies (*n* = 22) versus non-responders who did not (*n* = 18). Supplementary Figure S2. Survival of non-responders based on level of TILs. Kaplan-Meier curves showing the **(A)** progression-free survival, and **(B)** overall survival of non-responders to anti-PD-1 based therapies with low TILs (*n* = 14) compared to patients with intermediate/high TILs (*n* = 23). Supplementary Figure S3. Levels of tumour-infiltrating lymphocytes in patients who are alive versus those who have died at the data cut. Bar graphs comparing the levels of intratumoural and peritumoural tumour-infiltrating lymphocytes between patients who were alive (*n* = 12) or dead (*n* = 25) at data cut-off. Error bars represent mean ± SD. Supplementary Figure S4. Summary of genomic and immune profiles of patients with innate resistance to anti-PD-1-based therapies. Schematic illustrating the clusters of patients based on their differentially expressed gene (DEG) expression profiles and expression of alternative immune drug targets (DT).
