# Supplementary material for: Clinical and Molecular Heterogeneity in Patients with Innate Resistance to Anti-PD-1 +/− Anti-CTLA-4 Immunotherapy in Metastatic Melanoma Reveals Distinct Therapeutic Targets
Source: Cancers (Basel). 2021 Jun 25;13(13):3186. doi: 10.3390/cancers13133186 (PMC8267740; doi:10.3390/cancers13133186)
Supplement: Supplementary file 1 [file cancers-13-03186-s001.zip › Supplementary Figures 1-4.pdf]

Supplementary Figure 1

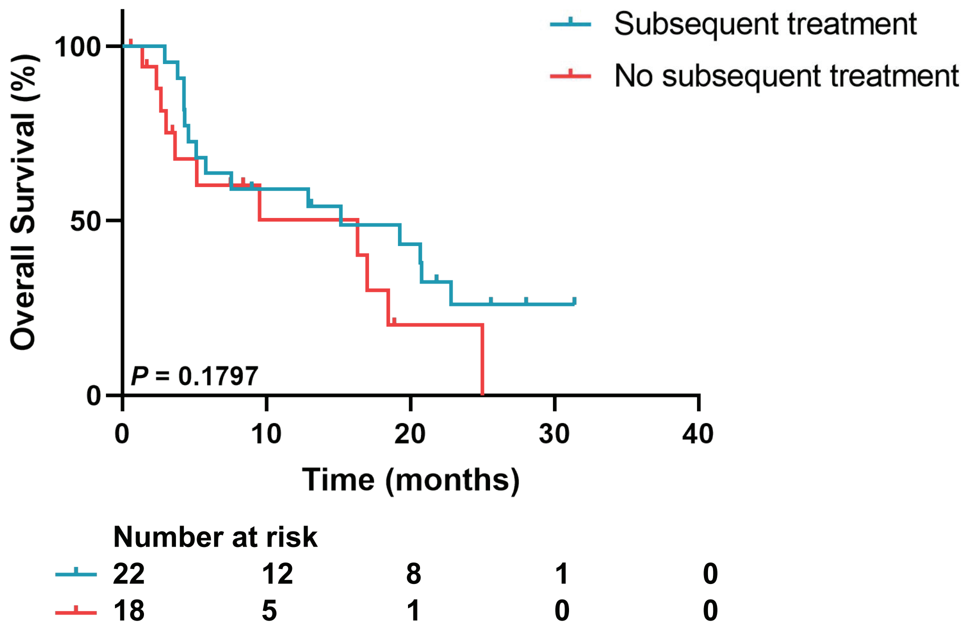

Supplementary Figure 2

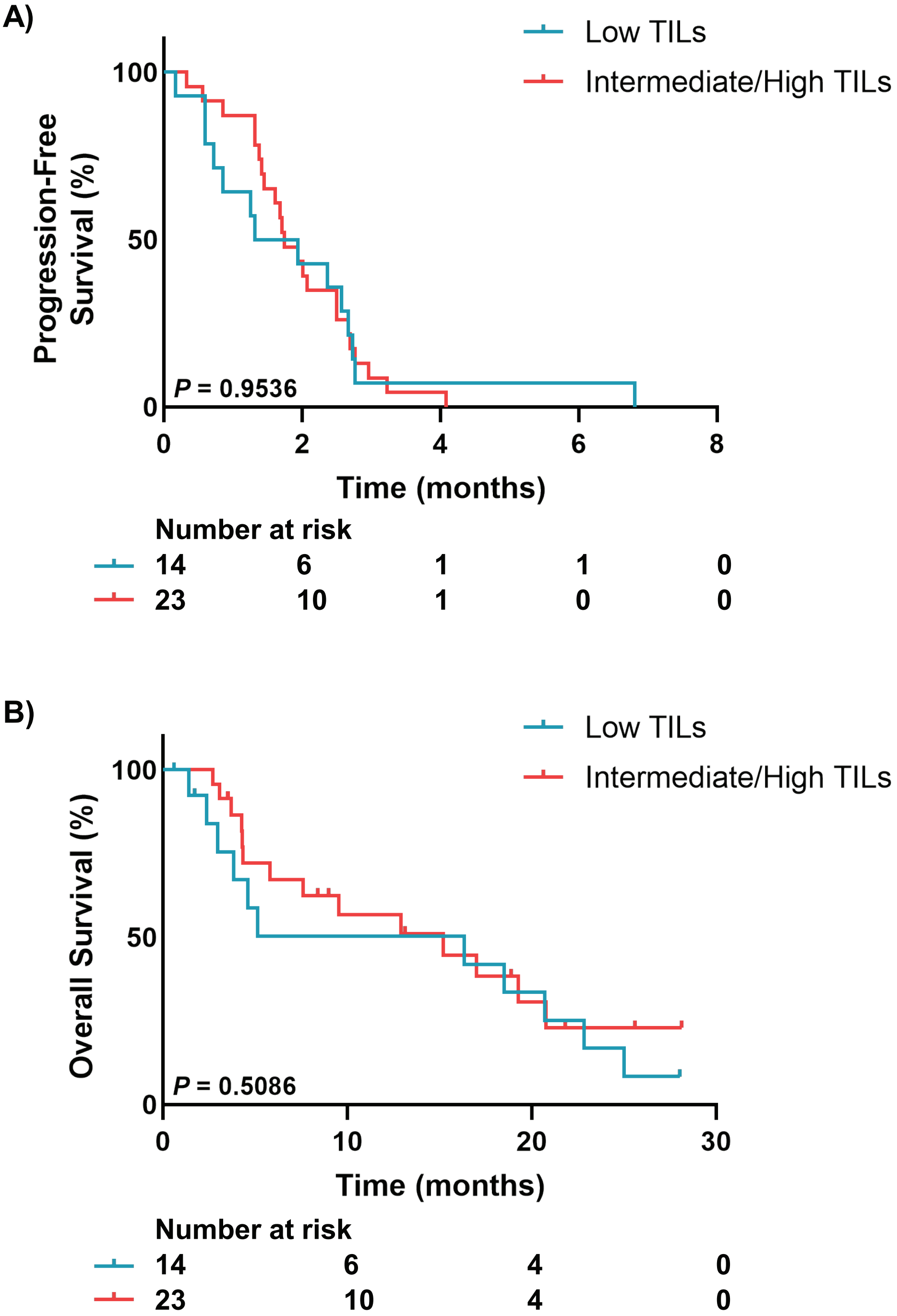

Supplementary Figure 3

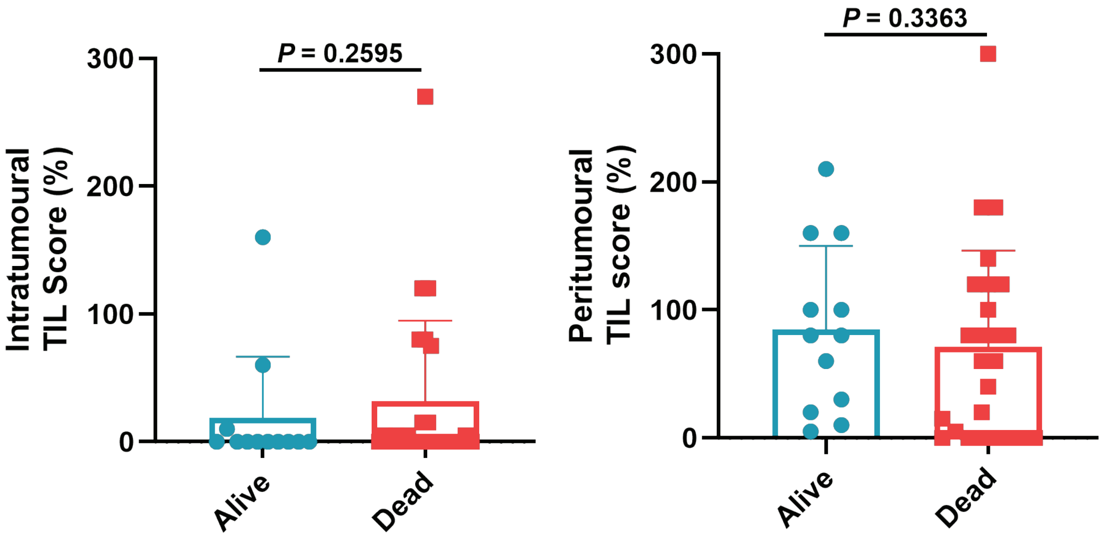

Supplementary Figure 4

DEG Cluster 1 + Int DT Cluster
- Genes associated with antigen presentation, immune response and IFN signalling
- Intermediate TILs and PD-L1 expression
- Intermediate expression of alternative drug targets (*ADA, CD276, PVR*)

DEG Cluster 2 + Int DT Cluster
- Genes associated with hypoxia, metabolism, signal transduction and neuronal signalling
- Intermediate TILs and PD-L1 expression
- Intermediate expression of alternative drug targets (*ADA, CD276, PVR*)

DEG Cluster 1 + High DT Cluster
- Genes associated with antigen presentation, immune response and IFN signalling
- High TILs and PD-L1 expression
- High expression of alternative drug targets (*IDO1, BTLA, ICOS, TIGIT*)

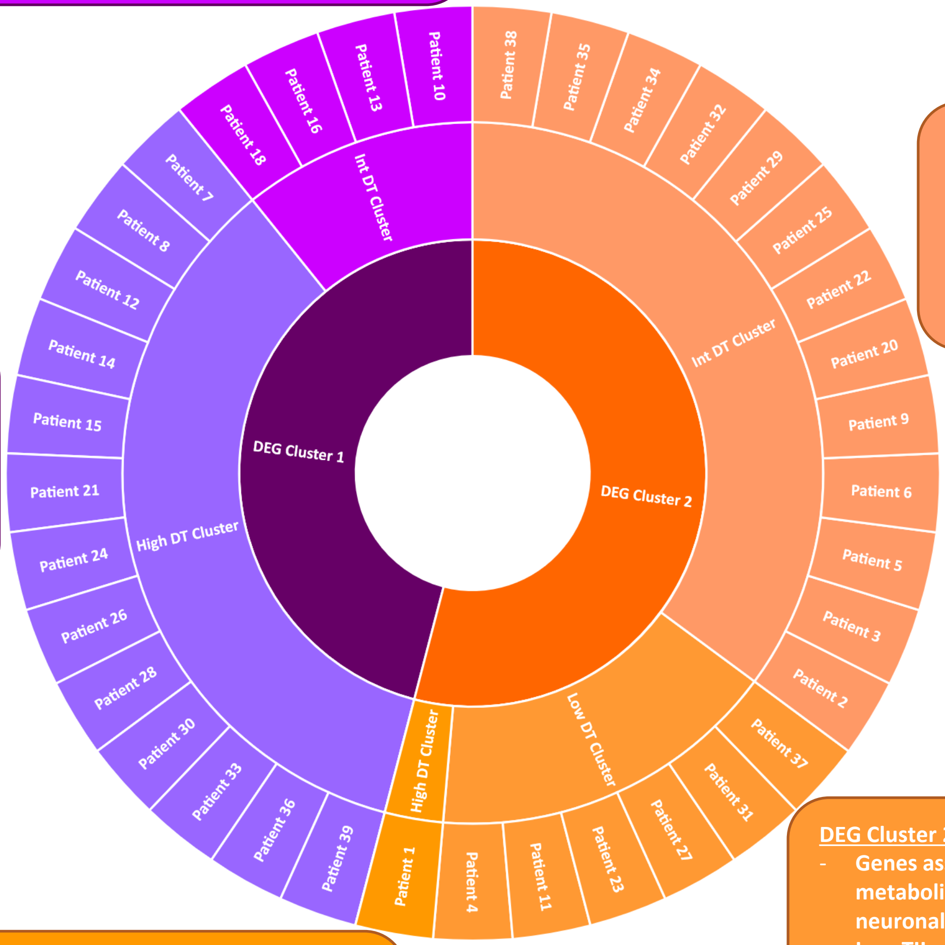

DEG Cluster 2 + Low DT Cluster
- Genes associated with hypoxia, metabolism, signal transduction and neuronal signalling
- Low TILs and PD-L1 expression
- Low expression of alternative drug targets

DEG Cluster 2 + High DT Cluster
- Genes associated with hypoxia, metabolism, signal transduction and neuronal signalling
- Low TILs and PD-L1 expression
- High expression of alternative drug targets (*IDO1, BTLA, ICOS, TIGIT*)
