# Supplementary material for: Clinical and Molecular Heterogeneity in Patients with Innate Resistance to Anti-PD-1 +/− Anti-CTLA-4 Immunotherapy in Metastatic Melanoma Reveals Distinct Therapeutic Targets
Source: Cancers (Basel). 2021 Jun 25;13(13):3186. doi: 10.3390/cancers13133186 (PMC8267740; doi:10.3390/cancers13133186)
Supplement: Supplementary file 1 [file cancers-13-03186-s001.zip › Supplementary Tables_3-9.pdf]

**Supplementary Table S3. Comparison of baseline clinical factors in rapid progressors (PFS < 2 months) versus other progressors (PFS ≥ 2 months).**

| Factor | PFS < 2 months (n=22) | PFS ≥ 2 months (n=18) | *P value* |
|---|---|---|---|
| **Age in years (median)** | 69 (34 – 96) | 65 (49 – 91) | 0.7984 |
| **Sex, male** | 16/22 (73%) | 10/18 (56%) | 0.3267 |
| **ECOG PS, ≥1** | 12/22 (55%) | 7/18 (39%) | 0.3596 |
| **Elevated LDH**[1] | 11/22 (50%) | 4/17 (24%) | 0.1116 |
| **Tumour burden** | **128 (19-366)** | **47 (15-200)** | **0.0625** |
| **Number of mets** | 6.5 (2-17) | 5 (1-12) | 0.2913 |
| **Number of organ sites with mets** | 3 (1-7) | 2 (1-4) | 0.1655 |
| **Brain mets** | 9/22 (41%) | 3/18 (17%) | 0.1654 |
| **Liver mets** | 8/22 (36%) | 3/18 (17%) | 0.2863 |
| **Staging, M1C/M1D** | 16/22 (73%) | 8/18 (44%) | 0.1064 |
| **BRAF V600E mutation**[2] | 5/8 (63%) | 0/1 (0%) | 0.4444 |

ECOG PS - Eastern Clinical Oncology Group (ECOG) performance status; LDH – lactate dehydrogenase; Tumor burden - sum of diameters of large metastases according to RECIST criteria; mets – metastases; n – number; % - percentage.

[1] missing values: 1

[2] as a proportion of total BRAF mutations

**Supplementary Table S4. Subsequent therapies received by patients who progressed on anti-PD-1-based therapies.**

| Subsequent Therapy | Progressed on anti-PD-1 (n=13) | Progressed on ipilimumab + anti-PD-1 (n=9) |
|---|---|---|
| **Ipilimumab + anti-PD-1** | 9/13 (69%) | 0/9 (0%) |
| **Ipilimumab monotherapy** | 3/13 (23%) | 0/9 (0%) |
| **Chemotherapy** | 1/13 (8%) | 2/9 (22%) |
| **Targeted therapy** | 0/13 (0%) | 7/9 (78%) |

**Supplementary Table S5. Comparison of baseline clinical factors in patients who are still alive versus those who have died at the data cut.**

| **Factor** | **Alive (n=14)** | **Dead (n=26)** | ***P value*** |
|---|---|---|---|
| **Age in years (median)** | 77 (37 – 96) | 65 (34 – 89) | 0.1539 |
| **Sex, male** | 9/14 (65%) | 17/26 (64%) | >0.9999 |
| **ECOG PS, ≥1** | 7/14 (50%) | 12/26 (46%) | >0.9999 |
| **Elevated LDH**[1] | 4/13 (31%) | 11/26 (42%) | 0.7281 |
| **Tumour burden** | 44 (15-366) | 121 (15-242) | 0.2741 |
| **Number of mets** | 5 (2-17) | 7 (1-12) | 0.4633 |
| **Number of organ sites with mets** | 2 (1-7) | 3 (1-5) | 0.4273 |
| **Brain mets** | 5/14 (36%) | 7/26 (27%) | 0.7201 |
| **Liver mets** | **1/14 (7%)** | **10/26 (38%)** | **0.0614** |
| **Staging, M1C/M1D** | 7/14 (75%) | 17/26 (65%) | 0.5001 |
| **BRAF V600E mutation**[2] | 2/5 (40%) | 3/4 (75%) | 0.5238 |

ECOG PS - Eastern Clinical Oncology Group (ECOG) performance status; LDH – lactate dehydrogenase; Tumor burden - sum of diameters of large metastases according to RECIST criteria; mets – metastases; n – number; % - percentage.

[1] missing values: 1

[2] as a proportion of total BRAF mutations

**Supplementary Table S6. Comparison of baseline clinical factors in non-responders with low TILs versus intermediate/high TILs.**

| **Factor** | **Low TILs (n=14)** | **Intermediate/High TILs (n=23)** | ***P value*** |
|---|---|---|---|
| **Age in years (median)** | 72 (49 – 96) | 67 (34 – 87) | 0.2192 |
| **Sex, male** | 10/14 (71%) | 14/23 (61%) | 0.7245 |
| **ECOG PS, ≥1** | 7/14 (50%) | 10/23 (43%) | 0.7447 |
| **Elevated LDH[1]** | 5/14 (36%) | 9/22 (41%) | >0.9999 |
| **Tumour burden** | 102 (19-202) | 118 (15-366) | 0.6111 |
| **Number of mets** | 7.5 (2-17) | 5.5 (2-17) | 0.8401 |
| **Number of organ sites with mets** | 2.5 (1-5) | 3 (1-7) | 0.9033 |
| **Brain mets** | 2/14 (14%) | 8/23 (35%) | 0.2603 |
| **Liver mets** | 6/14 (43%) | 5/23 (22%) | 0.2679 |
| **Staging, M1C/M1D** | 7/14 (50%) | 15/23 (65%) | 0.4933 |
| **BRAF V600E mutation[2]** | 1/3 (33%) | 4/5 (80%) | 0.4643 |

ECOG PS - Eastern Clinical Oncology Group (ECOG) performance status; LDH – lactate dehydrogenase; Tumor burden - sum of diameters of large metastases according to RECIST criteria; mets – metastases; n – number; % - percentage.

[1] missing values: 1

[2] as a proportion of total BRAF mutations

**Supplementary Table S7. KEGG pathway analysis of differentially expressed genes.**

| NAME | SIZE | ES | NES | NOM p-val | FDR q-val |
|---|---|---|---|---|---|
| **KEGG_HEMATOPOIETIC_CELL_LINEAGE** | 3 | -0.82609 | -1.50926 | 0.027896997 | 0.10045286 |
| **KEGG_PRIMARY_IMMUNODEFICIENCY** | 2 | -0.83333 | -1.35503 | 0.104803495 | 0.17845923 |
| **KEGG_T_CELL_RECEPTOR_SIGNALING_PATHWAY** | 2 | -0.83333 | -1.33643 | 0.092337914 | 0.16044548 |

**Supplementary Table S8. Comparison of baseline clinical factors between non-responders in Cluster 1 and Cluster 2 based on unsupervised hierarchical clustering of 26 differentially expressed genes.**

| Factor | RNA cluster 1 (n=17) | RNA cluster 2 (n=20) | *P value* |
|---|---|---|---|
| **Age in years (median)** | 61 (33 – 86) | 70 (41 – 95) | 0.1174 |
| **Sex, male** | 12/17 (71%) | 12/20 (60%) | 0.7307 |
| **ECOG PS, ≥1** | 5/17 (29%) | 12/20 (60%) | 0.0994 |
| **Elevated LDH**[1] | 5/16 (31%) | 9/20 (45%) | 0.5007 |
| **Tumour burden**[2] | 118 (15 – 242) | 90.5 (15 – 366) | 0.5452 |
| **Number of mets**[2] | 5.5 (2 – 12) | 7 (1 – 17) | 0.7297 |
| **Number of organ sites with mets** | 3 (1-4) | 2 (1-7) | 0.5363 |
| **Brain mets** | 4/17 (24%) | 6/20 (30%) | 0.7246 |
| **Liver mets** | 5/17 (29%) | 6/20 (30%) | >0.9999 |
| **Staging, M1C/M1D** | 11/17 (65%) | 11/20 (55%) | 0.7384 |
| **BRAF V600E mutation**[3] | 2/3 (67%) | 3/5 (60%) | >0.9999 |
| **Received subsequent therapies** | 10/17 (59%) | 11/20 (55%) | >0.9999 |

ECOG PS - Eastern Clinical Oncology Group (ECOG) performance status; LDH – lactate dehydrogenase; Tumor burden - sum of diameters of large metastases according to RECIST criteria; mets – metastases; n – number; % - percentage.

[1] missing values: 1

[2] missing values: RNA cluster 1 – 1; RNA cluster 2 – 4

[3] as a proportion of total BRAF mutations

**Supplementary Table S9. Comparison of baseline clinical factors between non-responders in Cluster A, Cluster B and Cluster C based on unsupervised hierarchical clustering of 36 genes, including druggable immune checkpoints.**

| Factor | RNA cluster A (n=6) | RNA cluster B (n=14) | RNA cluster C (n=17) | *P value* |
|---|---|---|---|---|
| **Age in years (median)** | 68 (51 – 77) | 63 (33 – 95) | 68 (41 – 88) | 0.8079 |
| **Sex, male** | 3/6 (50%) | 9/14 (64%) | 12/17 (71%) | 0.7355 |
| **ECOG PS, ≥1** | 4/6 (67%) | 4/14 (29%) | 9/17 (53%) | 0.2291 |
| **Elevated LDH[1]** | 2/6 (33%) | 2/13 (15%) | 10/17 (59%) | **0.0541** |
| **Tumour burden[2]** | 137 (15 – 166) | 76 (15 – 242) | 122 (19 – 366) | 0.9026 |
| **Number of mets[2]** | 8 (3 – 9) | 5 (2 - 12) | 6.5 (1 – 17) | 0.6626 |
| **Number of organ sites with mets** | 2 (1-4) | 3 (1-4) | 3 (1-7) | 0.4772 |
| **Brain mets** | 1/6 (17%) | 3/14 (21%) | 6/17 (35%) | 0.6116 |
| **Liver mets** | 1/6 (17%) | 3/14 (21%) | 7/17 (41%) | 0.3963 |
| **Staging, M1C/M1D** | 2/6 (33%) | 8/14 (57%) | 12/17 (71%) | 0.27 |
| **BRAF V600E mutation[3]** | 1/2 (50%) | 1/2 (50%) | 3/4 (75%) | >0.9999 |
| **Received subsequent therapies** | 3/6 (50%) | 7/14 (50%) | 11/17 (65%) | 0.6709 |

ECOG PS - Eastern Clinical Oncology Group (ECOG) performance status; LDH – lactate dehydrogenase; Tumor burden - sum of diameters of large metastases according to RECIST criteria; mets – metastases; n – number; % - percentage.

[1] missing values: 1

[2] missing values: RNA cluster A – 1; RNA cluster B – 1; RNA cluster C – 1

[3] as a proportion of total BRAF mutations
